# Real-time COVID-19 detection via graphite oxide-based field-effect transistor biosensors decorated with Pt/Pd nanoparticles

**DOI:** 10.1038/s41598-022-22249-2

**Published:** 2022-10-28

**Authors:** Asma Wasfi, Falah Awwad, Naser Qamhieh, Badria Al Murshidi, Abdul Rasheed Palakkott, Juri George Gelovani

**Affiliations:** 1 Department of Electrical and Communication Engineering, College of Engineering, United Arab Emirates University, P. O. Box 15551, Al Ain, United Arab Emirates; 2 Department of Physics, College of Science, United Arab Emirates University, P.O. Box 15551, Al Ain, United Arab Emirates; 3 Department of Biology, College of Science, United Arab Emirates University, P.O. Box 15551, Al Ain, United Arab Emirates; 4 College of Medicine and Health Sciences, United Arab Emirates University, Al Ain, United Arab Emirates; 5 Zayed Center for Health Sciences, United Arab Emirates University, Al Ain, United Arab Emirates

**Keywords:** Engineering, Nanoscience and technology

## Abstract

Coronavirus 2019 (COVID-19) spreads an extremely infectious disease where there is no specific treatment. COVID-19 virus had a rapid and unexpected spread rate which resulted in critical difficulties for public health and unprecedented daily life disruption. Thus, accurate, rapid, and early diagnosis of COVID-19 virus is critical to maintain public health safety. A graphite oxide-based field-effect transistor (GO-FET) was fabricated and functionalized with COVID-19 antibody for the purpose of real-time detection of COVID-19 spike protein antigen. Thermal evaporation process was used to deposit the gold electrodes on the surface of the sensor substrate. Graphite oxide channel was placed between the gold electrodes. Bimetallic nanoparticles of platinum and palladium were generated via an ultra-high vacuum (UHV) compatible system by sputtering and inert-gas condensation technique. The biosensor graphite oxide channel was immobilized with specific antibodies against the COVID-19 spike protein to achieve selectivity and specificity. This technique uses the attractive semiconductor characteristics of the graphite oxide-based materials resulting in highly specific and sensitive detection of COVID-19 spike protein. The GO-FET biosensor was decorated with bimetallic nanoparticles of platinum and palladium to investigate the improvement in the sensor sensitivity. The in-house developed biosensor limit of detection (LOD) is 1 fg/mL of COVID-19 spike antigen in phosphate-buffered saline (PBS). Moreover, magnetic labelled SARS-CoV-2 spike antibody were studied to investigate any enhancement in the sensor performance. The results indicate the successful fabrication of a promising field effect transistor biosensor for COVID-19 diagnosis.

## Introduction

The 2019–2020 outbreak of COVID-19 virus resulted in thousands of deaths and in worldwide panic^1^. The COVID-19 virus is extremely contagious. COVID-19 is not only a health crisis^2^, but it is also causing an economic burden^1^. COVID-19 was announced as a global health emergency and pandemic in March 2020^3^. World Health Organization (WHO) recommended performing diagnostic tests to stop the COVID-19 transmission and reduce the number of cases. COVID-19 pandemic is considered as a global challenge and researchers are working on vaccines, medicines, and detection mechanisms. Testing is critical for life-saving treatment and isolations of patients to prevent the spread of COVID-19 disease. Biosensing is crucial to detect and control such diseases. Quick diagnostic techniques are critical to reduce and prevent the virus spread rate.

One of the critical challenges in fighting COVID-19 is the accurate and rapid identification of infected patients including asymptomatic patients. Identifying those patients helps in applying protective measures such as lockdown and isolating patients which will result in slowing down the transmission rate of the infection. This is an important step for hospitals to provide sufficient supplies, doctors, and rooms to successfully treat the patients who need care. Therefore, diagnostic tests are critical to control the spread of COVID-19 disease. There is a need for new biosensors and diagnostic tests that are accurate, sensitive, reliable, rapid, and cheap. These biosensors should be capable of real-time, and label free identification of the virus in samples without the need for sample preparation. Biosensors based on semiconductor field-effect devices (FEDs) are one of the most promising platforms for an electrical identification of charged biomolecules.

The essential detection mechanisms of COVID-19 are categorized in three groups: antibody testing, nucleic acid detection, and chest computed tomography (CT) scan^4–8^. Various rapid detection methods are developed which is based on antigen and antibody sensors^1,5,9,10^. Real time polymerase chain reaction (PCR) is the test recommended by WHO to detect COVID-19 virus^9,11,12^. However, PCR requires long processing time, trained personal, and expensive equipment. The long time needed for PCR is due to the amplification steps before the detection process. The time required for the amplification process can be reduced by using a highly sensitive and selective detection process. Although researchers are continuously exploring rapid and accurate techniques to detect COVID-19, there is still a critical need to find a fast, user-friendly, and affordable testing technique to detect COVID-19.

Various biosensors have been developed and tested for the detection of COVID-19 virus. Moitra et al., used N gene-targeted antisense oligonucleotide-capped plasmonic nanoparticles and a colorimetric test to detect SARS-CoV-2N gene^13^. Qui et al., used two-dimensional gold nanoislands (AuNIs) functionalized with complementary DNA and localized surface plasmon resonance (LSPR) to detect SARS-CoV-2 genes^14^. Other detection techniques include nanopore sequencing^15^, biosensors based on cell^16^, and gel card agglutination tests^17^.

Biosensing aims to identify biochemical and biological targets by using biological elements in a chemical reaction, such as enzyme biosensors, or via binding to the required molecule^18,19^. This kind of binding can be converted to a measurable signal^20^. Similarly, graphene-based field-effect transistor biosensors have been designed, fabricated, and functionalized with specific antibodies to detect COVID-19 in clinical nasopharyngeal samples^10^. Moreover, graphene-based field-effect transistor functionalized with phosphorodiamidate morpholino oligos (PMO) and decorated with gold nanoparticles (AuNP) was used for unamplified and rapid detection of COVID-19^5^.

The most critical step during the fabrication of immunosensors, is the immobilization of the specific biomolecules such as antibodies. Therefore, nanomaterials are selected and considered as a substantial matric for the biomolecule’s immobilization. Biosensors based on nanomaterials has high surface to volume ratio which enhances the loading capacity of the sensing platform. Nanomaterials has various characteristics such as optical, thermal, electrical, and catalytic with intense mechanical strength. These characteristics show promising opportunities for the biosensors to identify infectious disease biomarkers. The large surface area of nanomaterials enhances the immobilization of bioreceptors by increasing the quantity and lowering the volume. Several nanomaterials were used such as graphene, nanotubes, gold, quantum dots, nanodiamonds, and polymeric nanoparticles^18,19^.

Among the various biosensing techniques, the field-effect transistor marked a unique presence due to its ability to detect small amounts of COVID-19 virus^3,9,21–23^. The field-effect transistor (FET) is a promising platform for the accurate and rapid detection of various biological targets^24–26^. The advantages of FET biosensors include low-cost, rapid response, and ease of use. FET biosensors can acquire high selectivity and sensitivity for specific target biomolecules by anchoring specific probes to the conducting channel which is highly important for FET sensor performance. Various semiconductor materials such as graphene, MoS_2_, and graphite oxide are used as conducting channels for FET biosensors due to their superior electronic characteristics^9,22^. Recently, graphene-based FET biosensors have been successfully utilized for the rapid detection of COVID-19 which triggered the scientific community’s interest in the current developments in graphene-based FETs^9,10^.

In this work, the designed GO-FET biosensor sensitivity was enhanced by using bimetallic nanoparticles of platinum and palladium and by utilizing magnetic labelled SARS-CoV-2 spike antibody. To the best of our knowledge, this is the first report that uses the pt/pd nanoparticles and the magnetic spike antibodies to analyze their influence on the biosensor performance.

Graphene is being used in the biosensing platform due to its unique properties^9,27,28^. Graphene is a monolayer of graphite and has a hexagonal lattice structure^27^. It is a two-dimensional sp^2^ hybridized form of carbon. Various techniques can be used to oxidize graphene and get graphene oxide (GO)^28^. Various synthesis methods can be used to get graphene and its derivatives such as graphene oxide or reduced graphene oxide^29–31^. The low production cost of graphene makes it an ideal material for the development of low-cost and high-performance sensors^28^. Graphene-based sensors are being widely popular nowadays^32^ and they are being used by researchers in various fields, such as viral and bacterial pathogens^33,34^ and vitamins^35^.

In this work, an electronic GO-FET biosensor was fabricated for real-time detection of COVID-19 spike antigen in solution. The GO-FET biosensor was decorated with bimetallic platinum/palladium nanoparticles for selective detection of the COVID-19 virus. Platinum/palladium nanoparticles were generated inside an ultra-high vacuum (UHV) compatible system by sputtering and inert-gas condensation technique; then they were self-assembled on top of the graphite oxide. After that, the graphite oxide channel was functionalized with SARS-CoV-2 spike antibody. The performance of this new COVID-19 biosensor was determined by measuring its dynamic response to the range of S-protein (spike protein) concentrations (1 fg/ml to 100 ng/ml). Moreover, magnetically-labeled antibodies to SARS-CoV-2 spike protein (250 μg/mL) were also tested to investigate any improvement in sensing.

## Materials and methods

### Chemicals and reagents

All chemicals and reagents were of the highest purity and analytical grade, including: SARS-CoV-2 (2019-nCoV) spike S1 His-tagged recombinant protein HPLC-verified (Catalog number: 40591-V08H; Sino Biological; Beijing, China); humanized monoclonal antibody against COVID-19 S1 protein (Catalog number: MBS8574745; My BioSource, San Diego, USA); rabbit anti-human IgG HRP-conjugated (Catalog number: ab6759; Abcam; Cambridge, UK); magnetic nanoparticles conjugation kit (Catalog number: ab269890; Abcam, Cambridge, UK); 1-Pyrenebutanoic Acid Succinimidyl Ester (PBASE) (Catalog number: P2767; Tokyo Chemical Industry, Tokyo, Japan).

### Coomassie blue staining and Western Blotting

For SDS-PAGE gel electrophoresis and Western blot, the spike protein samples were prepared following standard procedures as reported previously^36^. In brief, the spike protein samples were heated in the SDS sample loading buffer (0.375 M Tris pH 6.8, 12% SDS, 60% glycerol, 0.6 M DTT, 0.06% bromophenol blue) at 95 °C for 10 min. Thereafter, the samples were separated by electrophoresis in 10% SDS-PAGE. Following electrophoresis, the gel was stained with Coomassie blue stain and finally, images were acquired to check for the integrity of the protein. For Western blotting, the resolved spike protein on the gel was electroblotted onto PVDF membrane (Millipore, CA, USA) at 4 °C for 1 h at 100 V. Following transfer, the blots were blocked using 5% skimmed milk in Tris-buffered saline (0.1 M Tris, 0.9% NaCl) added with 0.1% Tween-20 (TBST) for 1 h at room temperature (RT) and concomitantly washed with TBST thrice. Thereafter, the membrane was incubated with Anti-COVID-19 antibody: Humanized COVID 19 Spike S1 Protein Coronavirus Monoclonal Antibody (1:1000) at 4 °C on an orbital shaker overnight. Following overnight incubation, the membrane was washed three times in TBST; and thereafter, incubated with secondary antibody Rabbit Anti-Human IgG (HRP) antibody (1:5000) for 1 h at RT on an orbital shaker and concomitantly washed with TBST thrice. Finally, protein bands were visualized using SuperSignal West Femto Maximum sensitivity substrate (Thermo Scientific, Logan, UT); and images were acquired using Image Studio Version 5.2 software from LI-COR Biosciences.

### Experiment setup

For the GO-FET biosensor fabrication, characterization, and testing; the following machines were utilized: (1) thermal evaporation system (Torr machine) to deposit the gold electrodes on the surface of the Silicon wafer, (2) Ultra-High Vacuum Compatible (UHV) system to generate the composite bimetallic nanoparticles, (3) Scanning Electron Microscope (SEM) to image the structure of the nanoparticles, (4) Energy-Dispersive X-Ray Spectroscopy (EDS) to confirm the nanoparticles composition, and (5) a computer-controlled Keithley 236 source-measuring system to generate current–voltage (I–V) measurements and test the sensor performance. The current was measured by measuring the ${I}_{d}$ with ${V}_{ds}$ sweeping from − 0.8 to 0.8 V. For the sensing process, ${I}_{d}$ was recorded with fixed ${V}_{ds} (0.1 V)$ while samples containing various concentrations of COVID-19 spike protein antigen were dropped on the biosensor channel. All the measurements were conducted at room temperature.

### Device fabrication

**Figure 1:**
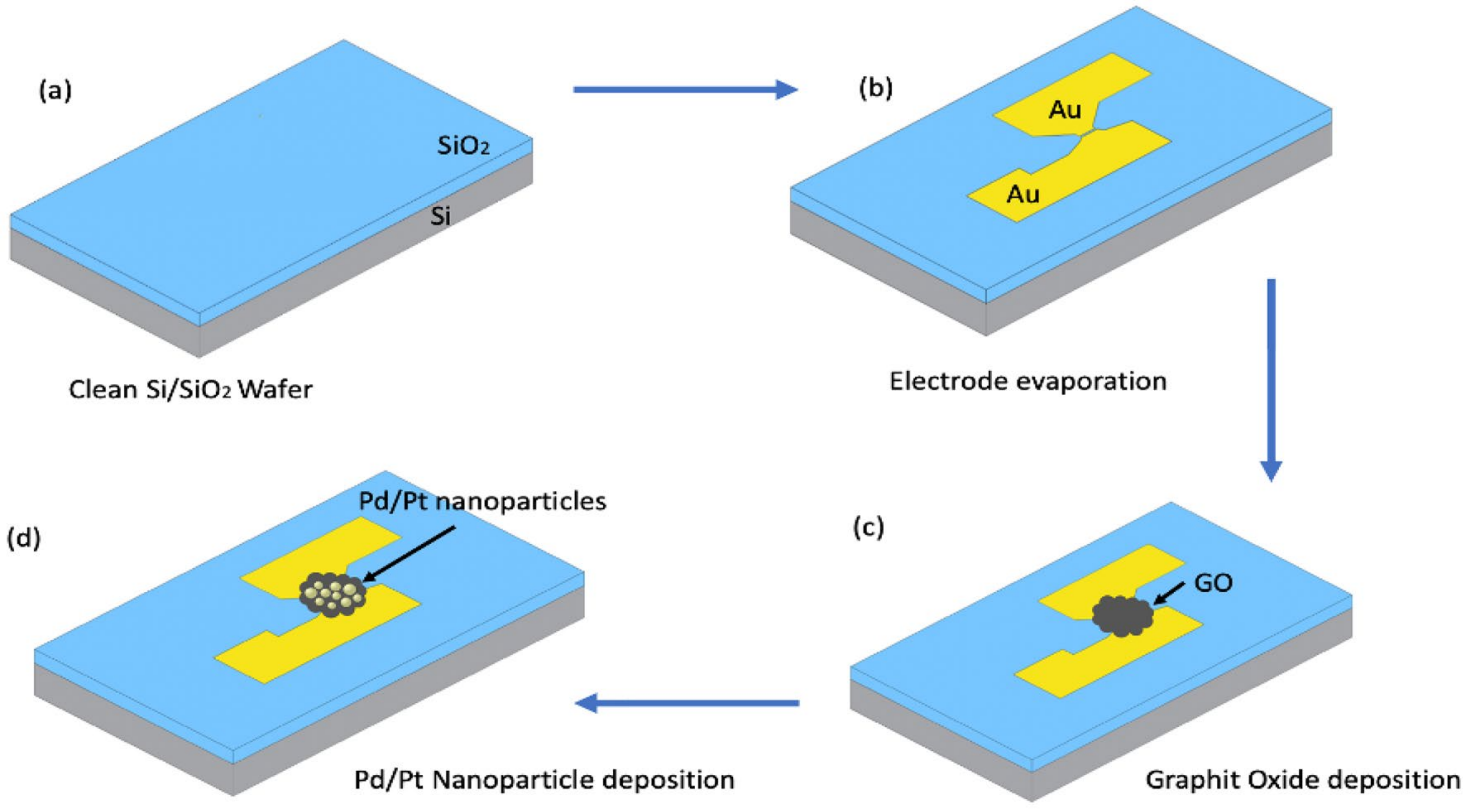
Schematic representation of the fabrication steps of the GO-FET biosensor. (**a**) The Si/SiO_2_ wafer was cleaned with acetone, ethanol, and deionized water. (**b**) Thermal evaporation was used to deposit the gold source and drain. (**c**) A graphite oxide drop was placed to form the channel. (**d**) Pd/Pt composite nanoparticles were sputtered on the graphite oxide channel using an Ultra High Vacuum compatible system (UHV).

COVID-19 transistor-based biosensor was fabricated by using a p-type silicon wafer (Si/SiO_2_) consisting of a p-type silicon layer (Si) and a silicon dioxide layer (SiO_2_) on top of it. The silicon wafer was cut into 1.0 cm × 0.5 cm pieces. Figure 1 illustrates the fabrication steps of the GO-FET biosensor. It started by using acetone, ethanol, and deionized water to clean the silicon wafer. Then, the wafer was dried using nitrogen gas. Thermal evaporation process was used to deposit the source and drain electrodes through the stainless-steel shadow mask. The electrodes consist of a 5 nm layer of Nickel-Chrome (NiCr) and a 50 nm layer of gold (Au) on top of it. The NiCr layer is used to improve the adhesion between the silicon wafer and the gold electrodes^37,38^. The FET channel was formed by depositing a drop of commercial graphite oxide (GO) of 4 mg/mL between the gold electrodes and leaving it for 24 h at room temperature to dry.

Composite nanoparticles of palladium and platinum were sputtered on the GO channel by using an ultra-high vacuum (UHV) compatible system (Nanogen-50, Mantis Deposition Ltd. Oxfordshire, UK). A composite target of palladium and platinum of 99.99% purity and ratio of 1:1 was fixed on the magnetron sputter head. Plasma was generated inside the source chamber using argon (Ar) inert gas. The plasma helps to sputter palladium/platinum nanoparticles from the target by using DC discharge power. The UHV system has a rotary pump and two turbo pumps which are used to reduce the pressure to 10^–6^ mbar^39^. Argon gas was utilized to generate plasma within the chamber to sputter metal nanoparticles from the Pd/Pt target by DC discharge power. Nanoparticles were generated and placed on the GO channel of the biosensor. The aggregation length (L) which is the distance between the exit nozzle and the target, was set at 80 mm. The discharge DC power was set at 128 W and the argon-gas flow rate was fixed at 70 sccm (standard cubic centimeter per minute). The nanoparticle size can be changed by modifying the aggregation length, the argon-gas flow rate, and the DC discharge power^40^.

### Immobilization of SARS-CoV-2 antibody on the surface of graphene biosensor

The fabricated graphene-based device was soaked in 2 mM PBASE (TCI, Tokyo, Japan) in methanol for 1 h at RT and then rinsed several times with PBS and DI water. Finally, the functionalized device was exposed to 250 μg/mL SARS-CoV-2 spike antibody for 3 h. The excess of antibody was removed by rinsing in PBS.

### Detection of the spike protein with the newly-developed graphene biosensor

Thereafter, to investigate the performance of the COVID-19 biosensor, we evaluated the dynamic response of this sensor to spike protein. To accomplish this, we tested different concentrations of the spike protein ranging from 1 fg/ml to 100 ng/ml. Also, we tested whether the improvement in sensitivity and dynamic range can be achieved by the addition of secondary magnetically labeled antibodies against SARS-CoV-2 spike protein (250 μg/mL) similar to a “sandwich” immunoassay. The magnetic nanoparticle labeling of antibodies against SARS-CoV-2 spike protein was accomplished according to the manufacturer-recommended protocol (Abcam, Cambridge, UK).

## Results and discussion

### Spike protein integrity and specificity of the antibody by Coomassie blue staining and Western Blotting

**Figure 2:**
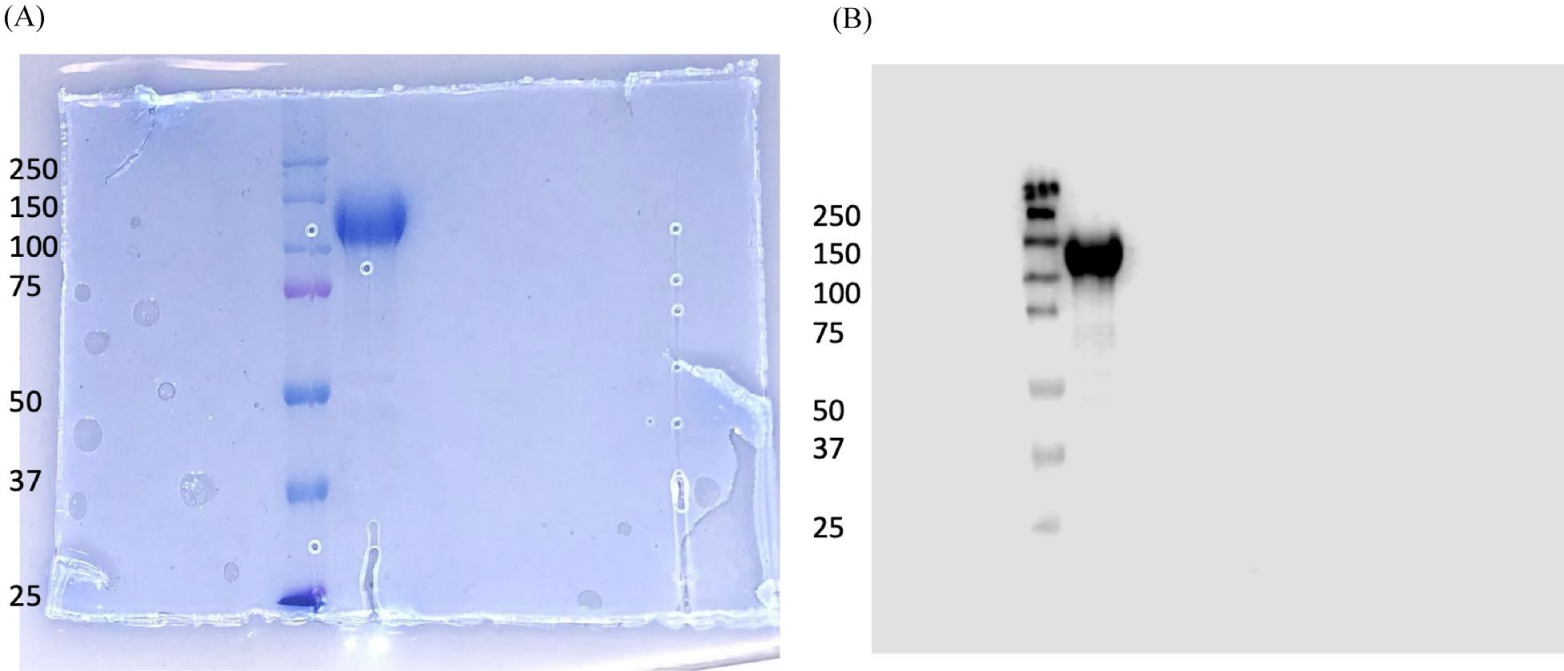
Analysis of the spike protein by Coomassie blue staining (**A**) and Western blot (**B**). Coomassie blue staining and Western blot of the gel confirmed the presence as well the integrity of the spike protein.

Figure 2 shows Coomassie staining and Western blot which confirms the presence and integrity of the spike protein. Coomassie staining and Western blot analysis of the gel revealed a clear band between 150 and 100 kDa, as expected. The presence of a single discrete band in between 150 and 100 kDa without any non-specific bands on the blot validates the antibody specificity used in the assay.

### Nanoparticle characterization

#### Size distribution of nanoparticles

**Figure 3:**
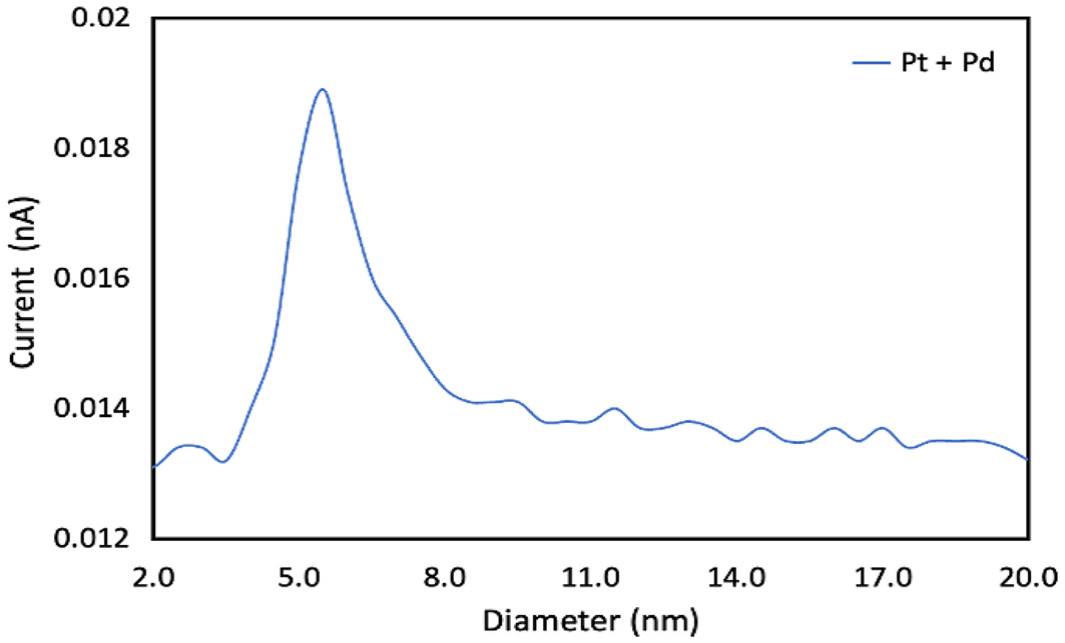
Size distribution of composite bimetallic (Pt and Pd) nanoparticles measured using QMF.

Figure 3 shows the size distribution for the bimetallic nanoparticles of platinum and palladium as measured using the QMF, where the average diameter was 5.5 nm.

#### SEM and EDS

**Figure 4:**
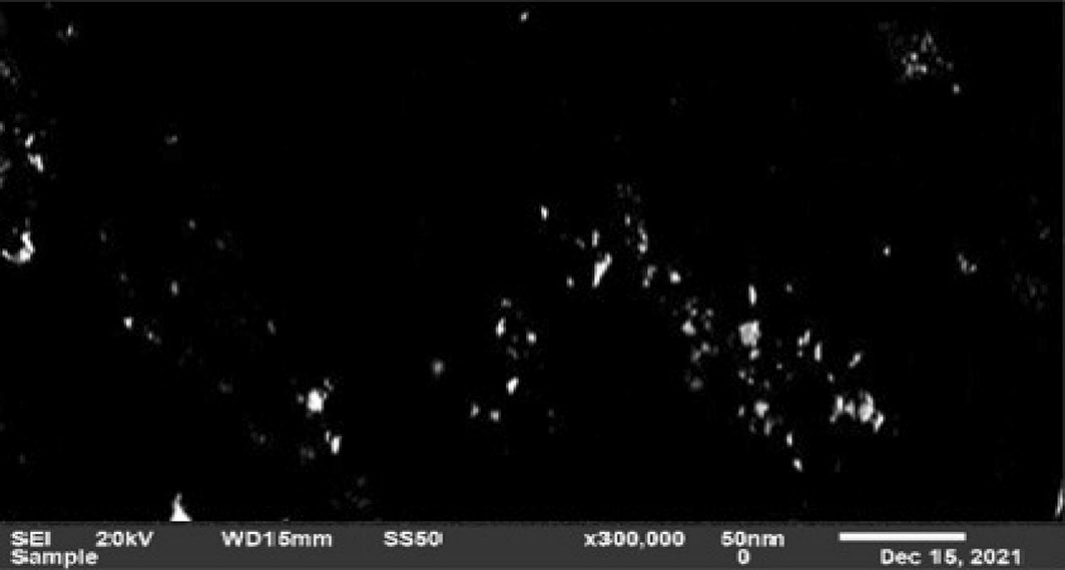
SEM image of bimetallic nanoparticles of palladium and platinum.

**Figure 5:**
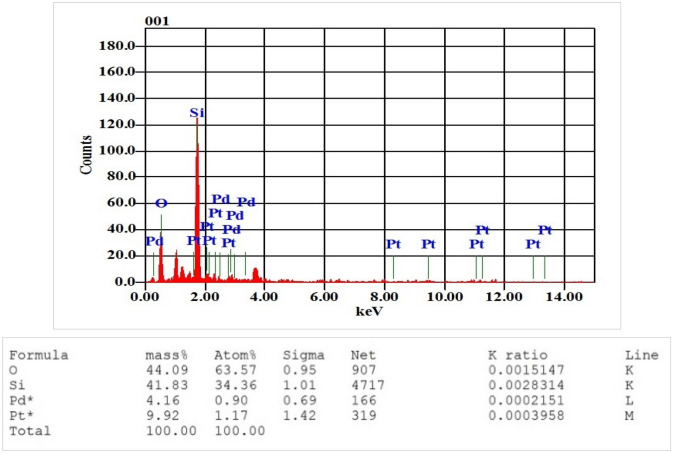
EDS spectrum of composite nanoparticles. The composite nanoparticles consist of palladium and platinum with mass percentage of 4.16% for palladium, and 9.92% for platinum. The existence of other elements such as silicon (Si), and Oxygen (O) is due to the glass substrate.

Figure 4 shows the scanning electron microscope (SEM) image of the bimetallic nanoparticles of platinum and palladium which reveals an agglomeration of the nanoparticles. The energy dispersive spectroscopy confirms the presence of platinum and palladium composite nanoparticles. Figure 5 shows the mass percentage of the two elements. The amount of mass percentage of palladium is 4.16% while platinum is 9.92%. The other elements that appeared in the EDS analysis such as oxygen and silicon are due to sputtering the nanoparticles on the glass substrate used for EDS analysis.

#### X-ray diffraction analysis (XRD)

**Figure 6:**
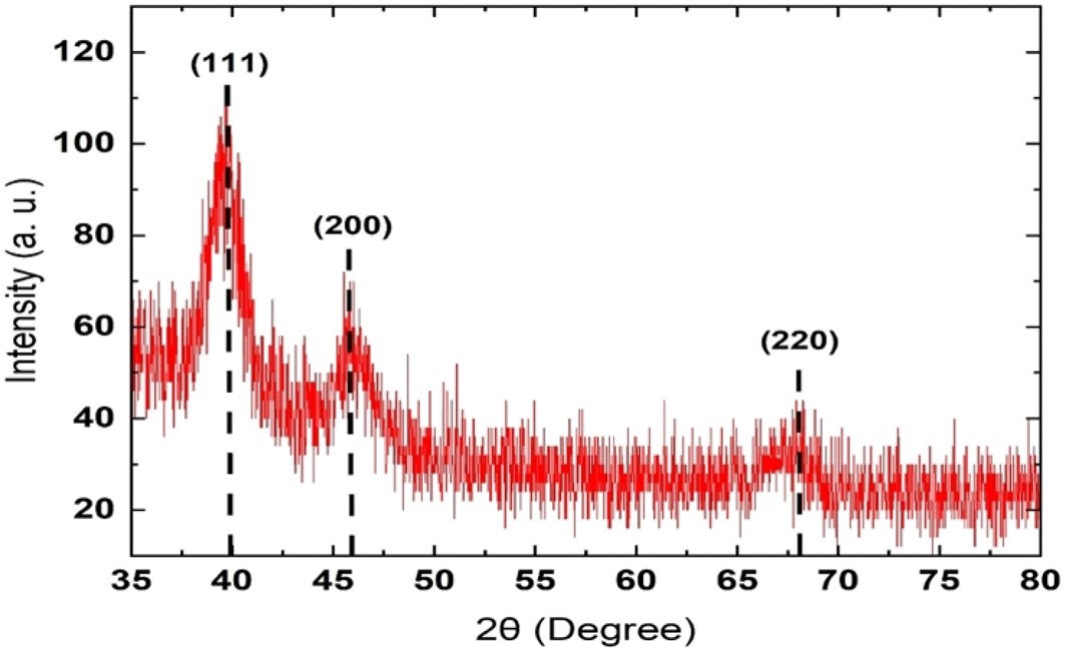
XRD pattern of the produced Pt/Pd composite nanoparticles.

The bimetallic nanoparticles characteristics are investigated by their size, morphology, structure, and the distribution of their elements. The X-ray diffraction (XRD) measurements of the bimetallic nanoparticles were generated to identify the structure of the bimetallic nanoparticles. Figure 6 shows the XRD pattern of Pt–Pd bimetallic nanoparticles. XRD was used to investigate the phase and structure of the generated Pd–Pt composite nanoparticles. The diffraction peaks at 2$\theta$ of 39.53°, 46.03°, and 67.86° correspond to diffractions from (111), (200), (220) planes of face-centered-cubic (fcc) structure which confirms the crystalline metallic nanoparticles^41–43^. The peeks formation between pure cubic palladium (0) (JCPDS 46-1043) and platinum (0) (JCPDS 04-0802) confirms the bimetallic nanoparticles of platinum and palladium alloy (JCPDF 65-6418).

### FET characteristics

**Figure 7:**
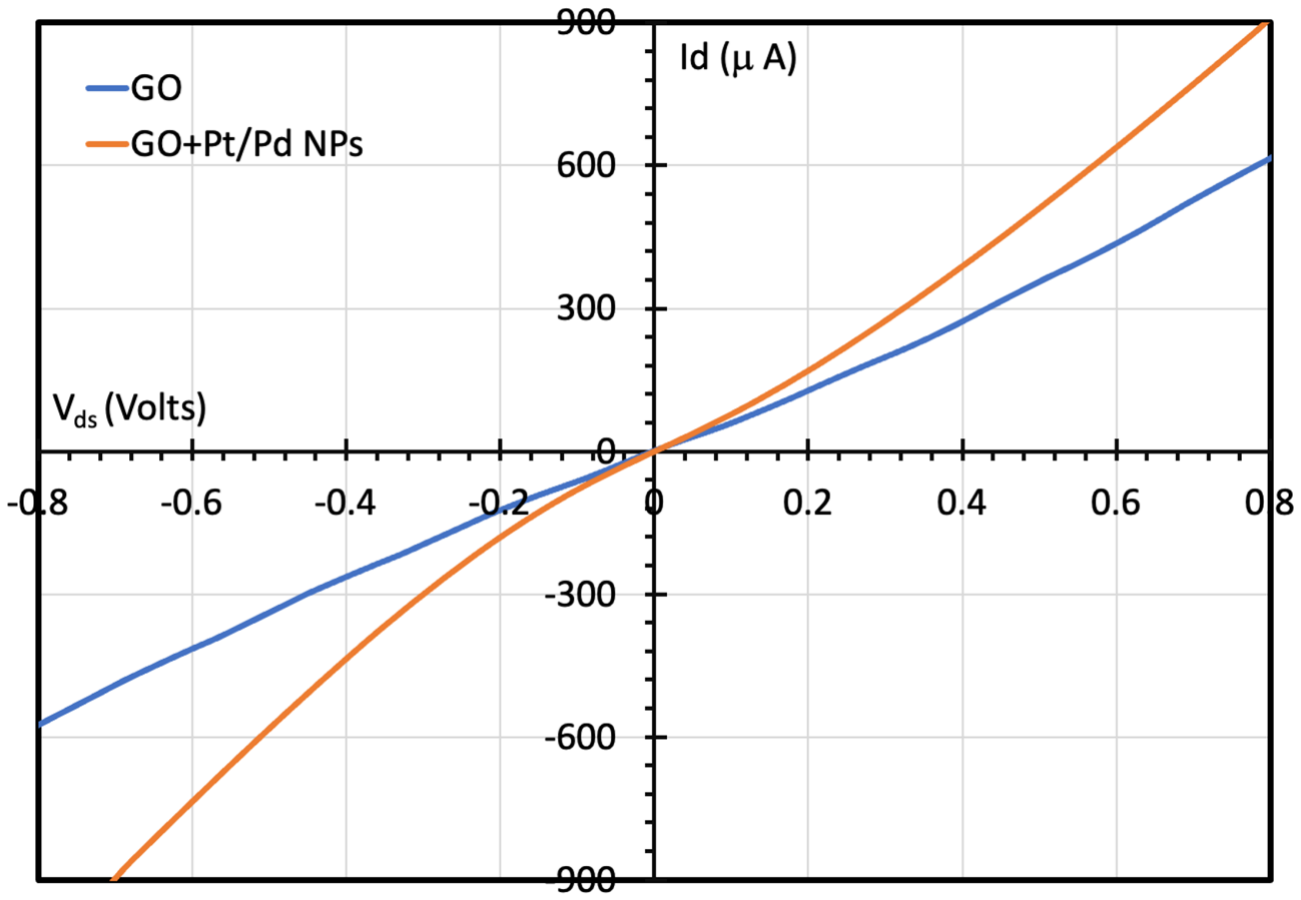
${I}_{d}-{V}_{ds}$ characteristics for both GO sensors: with and without bimetallic nanoparticles.

The GO-FET electrical characteristics were read at room temperature. Figure 7 shows the drain current (${I}_{d}$) versus drain-source voltage (${V}_{ds}$) curve for both the sensor without and with Pd/Pt nanoparticles. The figure shows a linear relationship between ${I}_{d}$ and ${V}_{ds}$ where the current increases by increasing the drain-source voltage. It was noticed that the addition of bimetallic nanoparticles resulted in a higher drain current at the same applied drain-source voltage since bimetallic nanoparticles of palladium and platinum exhibit higher conductivity as compared with graphite oxide.

### Sensing mechanism

**Figure 8:**
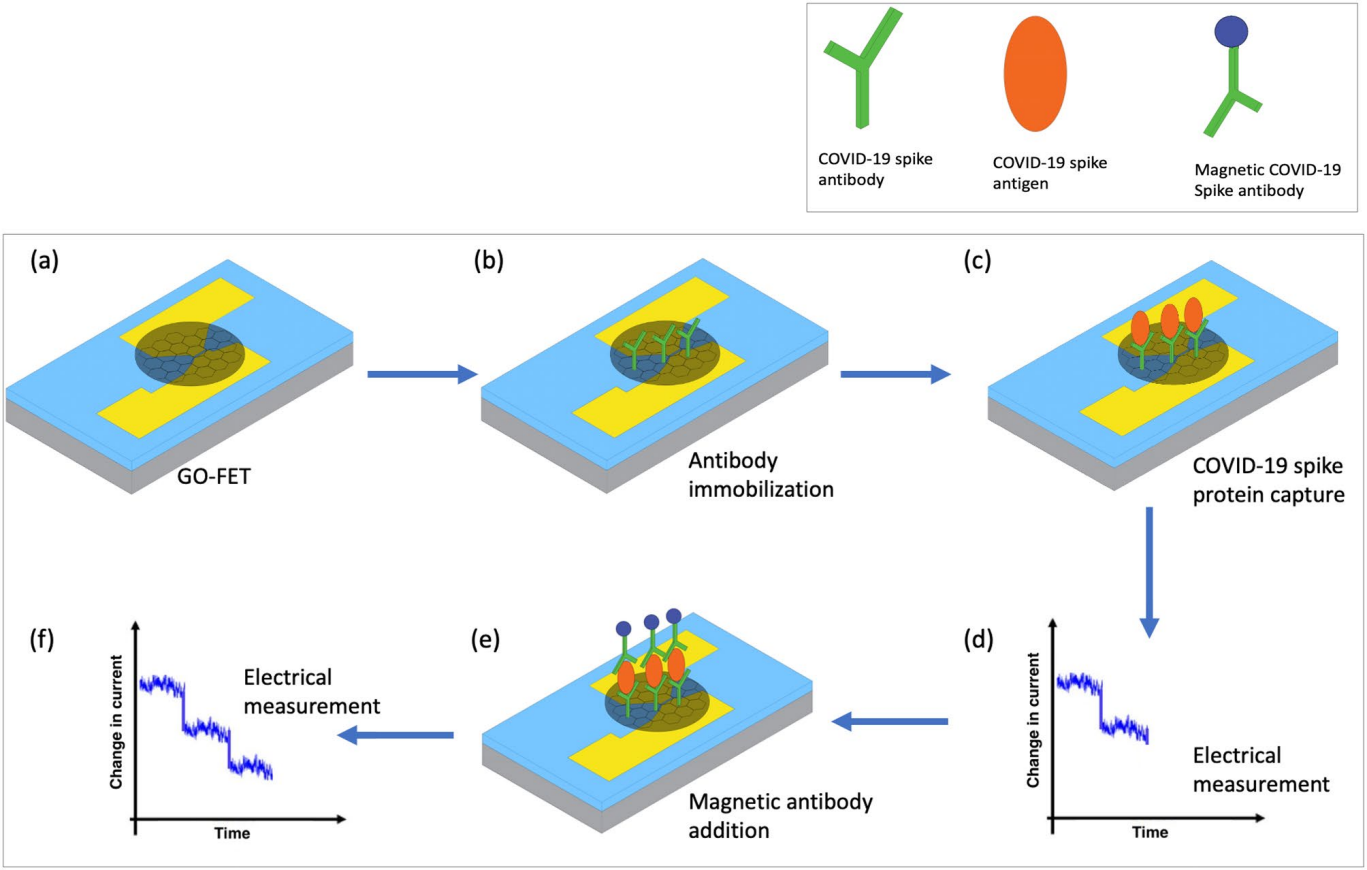
(**a**) Schematic diagram of the GO-FET biosensor. (**b**) COVID-19 spike protein antibody immobilized on the GO-FET channel. (**c**) Target COVID-19 spike protein captured by the biosensor. (d) The electrical current variation due to capturing the spike protein. (**e**) Magnetic COVID-19 spike protein antibody addition. (**f**) The electrical current variation for the GO-FET biosensor due to Magnetic COVID-19 spike protein addition. Sensor testing.

Figure 8 illustrates the working mechanism of the fabricated biosensor. Figure 8a shows the GO-FET where the electrodes are made of gold, while the channel is made of semiconducting graphite oxide. Figure 8b shows the COVID-19 spike protein antibody that is immobilized to the GO channel while Fig. 8c shows the target COVID-19 spike protein molecule captured by the immobilized antibodies. After that, Fig. 8d shows that when the negatively charged target molecule binds the GO-FET biosensor, a depletion of the charge carriers occurs leading to a decrement in the electrical current. Figure 8e shows that the addition of magnetic COVID-19 spike protein antibody leads to further decrement in electrical current as displayed in Fig. 8f.

**Figure 9:**
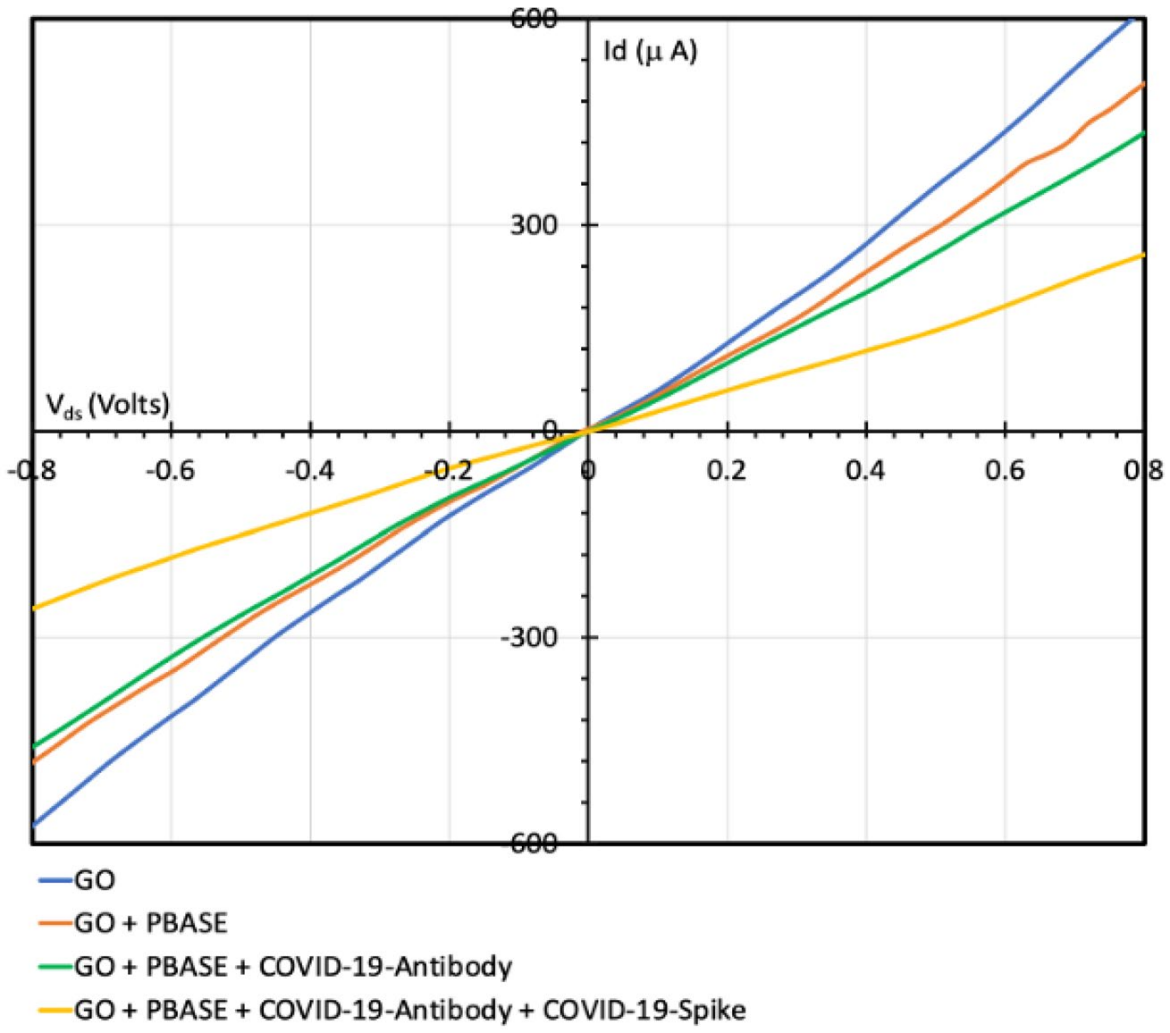
Electrical characterization of GO-FET, GO-FET modified with PBASE, GO-FET with COVID-19 spike antibody-immobilized on the GO channel, and the spike protein detection.

Figure 9 shows the current–voltage (I–V) curves of the GO-FET device over a range from − 0.8 to + 0.8 V before and after the antibody attachment. After functionalizing the GO channel with PBASE the slope (dI/dV) decreased. Moreover, the COVID-19 spike antibody immobilization led to a further decrement in the (dI/dV) slope. The (dI/dV) slope change indicates the successful addition of the COVID-19 spike antibody. Figure 9 shows that the highest decrement in slope is due to capturing the target COVID-19 spike protein. In each of the above steps a decrement in the GO-FET mobility occurred due to additional sources of dispersion of the charge carrier^44^. The decrement in ${I}_{d}$ is because of the accumulation of the negative charge carriers from the PBASE, the immobilized antibodies, and the binding between the antibodies and the COVID-19 spike protein. These results are consistent with previous studies^3,45^. Seo et al. was able to fabricate a graphene based FET for real time detection of COVID-19 spike protein^10^. The negatively charged COVID-19 spike protein binds with the antibody resulting in a decrement in the ${I}_{d}$ response.

COVID-19 virus consists of four structural proteins: spike, matrix, envelope, and nucleocapsid. Spike protein is considered the best to diagnose and detect the antigen because it is highly immunogenic and it is a major transmembrane protein of the virus. Thus, COVID-19 spike protein and COVID-19 spike antibody were used in this work to specifically detect the COVID-19 virus.

The GO-FET sensor was fabricated with a graphite oxide channel conjugated with a COVID-19 spike antibody, where the FET channel was covered with 4 µL of 2 mM of phosphate-buffered saline. After that, the GO-FET was exposed to 250 μg/mL of COVID-19 spike antibody for 3 h to investigate the performance of GO-FET biosensors. The GO-FET channel was functionalized either with MERS spike protein antibodies or with COVID-19 spike protein antibodies.

**Figure 10:**
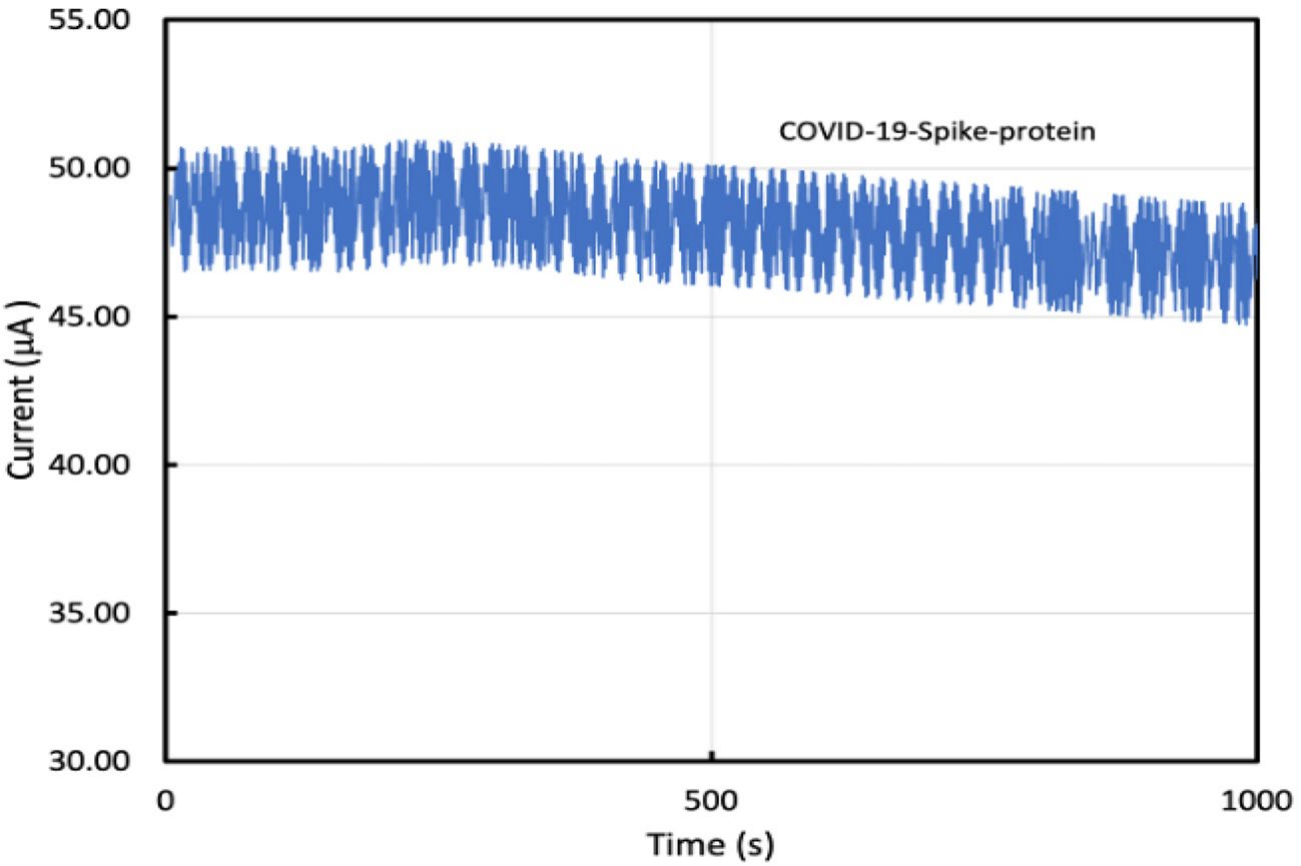
Variation in the drain current due to 0.4 μL drop of 1 pg/mL of COVID-19 spike antigen for GO-FET biosensor functionalized with Middle East Respiratory Syndrome (MERS) antibody.

**Figure 11:**
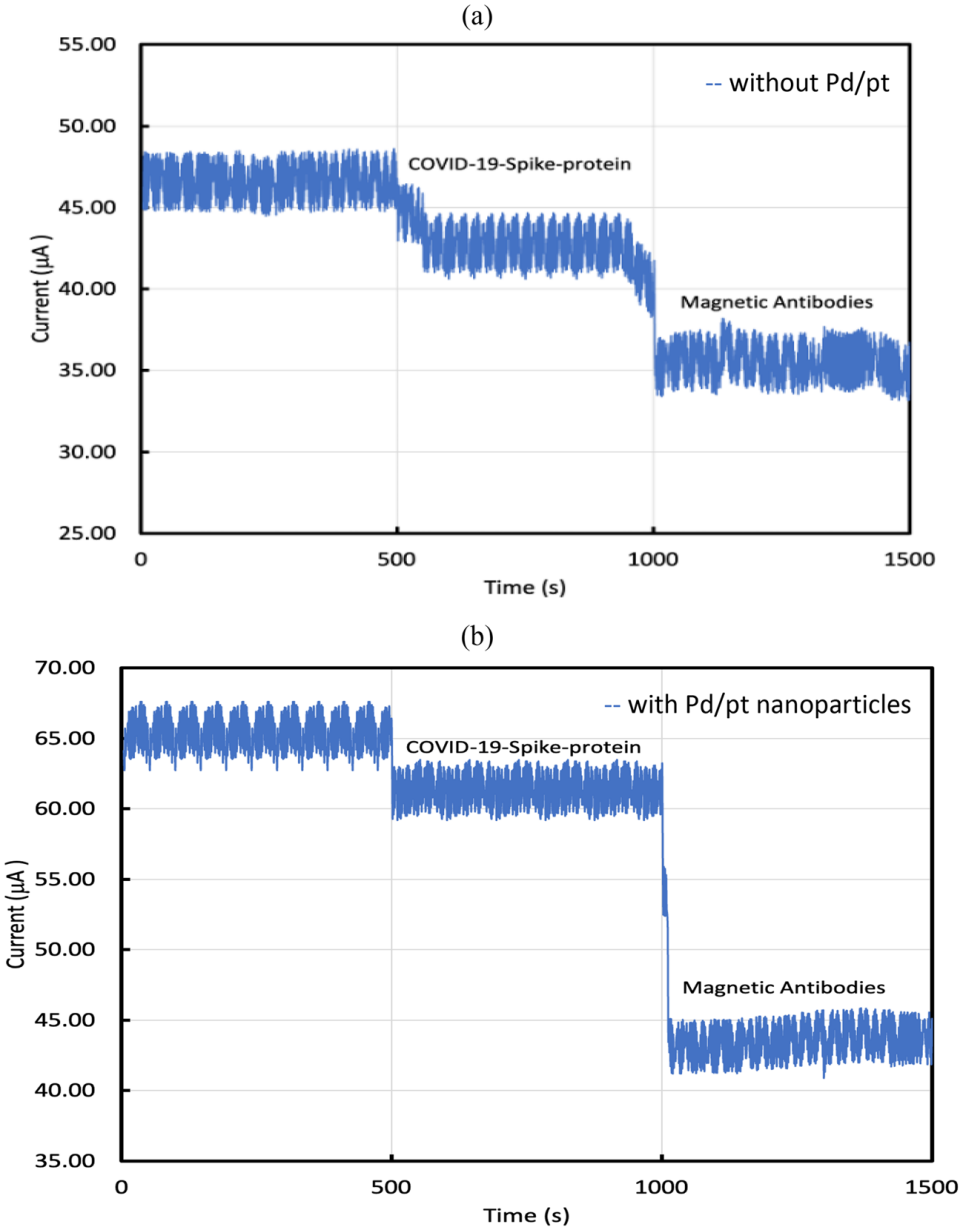
Variation in the drain current due to a 0.4 μL drop of 1 pg/mL of COVID-19 spike antigen for GO-FET biosensor functionalized with COVID-19 spike antibody (**a**) without nanoparticles, and (**b**) with Pd/Pt nanoparticles.

Figure 10 shows that the GO-FET functionalized with MERS antibodies didn’t show any remarkable variation of electrical current due to the addition of COVID-19 spike antigen. While Fig. 11 shows that GO-FET functionalized with COVID-19 spike antibodies was able to detect COVID-19 spike antigen. Thus, COVID-19 spike antibodies are essential for specific binding with COVID-19 spike antigen. The biosensor functionalized with MERS antibodies exhibited no response to COVID-19 spike protein, indicating that COVID-19 functionalized with COVID-19 spike antibodies is specific and sensitive to COVID-19 spike antigen.

The GO-FET biosensor functionalized with COVID-19 spike antibodies was tested for different concentrations of COVID-19 spike antigen. Figure 11a displays the current variation (at ${V}_{ds}$ = 0.1 V) when the graphite oxide channel was exposed to 0.4 μL drop of 1 pg/mL of COVID-19 spike antigen. Figure 11a displays the current of the biosensor without Pd/Pt nanoparticles where the current started at ${I}_{max}$= 46.5 μA when the 0.4 μL drop of 1 pg/mL of COVID-19 spike antigen was added at time 500 s, the current drop was noticed and reached ${I}_{min}$= 42.3 μA. The variation in current was utilized to evaluate the sensor performance and sensitivity. The current variation was calculated as ${I}_{max}-{I}_{min}$. Moreover, Magnetic COVID-19 antibodies were added to investigate their effect on the biosensor performance. Figure 11a shows a further drop in current due to the addition of 5 μL of magnetically labeled anti-COVID-19 spike antibodies (250 μg/mL) at time = 1000 s. The additional drop of the current at time 1000 s is because of the accumulation of the negative charge carriers which confirms that the magnetically-labeled antibodies improved the sensitivity. The current ${I}_{d}$ reached 35.54 μA as the result of the addition of magnetically-labeled antibodies. Thus, magnetic antibodies enhanced the sensor performance and sensitivity.

Moreover, the GO-FET biosensor decorated with bimetallic nanoparticles of platinum and palladium and functionalized with COVID-19 spike antibodies was studied as displayed in Fig. 11b. Figure 11b shows the variation of current for the biosensor decorated with bimetallic nanoparticles. The biosensor current started at ${I}_{max}$ = 65.2 μA and when the COVID-19 spike antigen was dropped on the sensor channel at time $t$= 500 s, the current ${I}_{d}$ drop was observed and reached ${I}_{min}$ = 60.9 μA. Moreover, when the magnetic antibodies were added at time = 1000 s, the current ${I}_{d}$ drop was observed and reached ${I}_{min}$ = 43.54 μA.

It was observed that the biosensor starting current with nanoparticles is higher than the biosensor starting current without nanoparticles. Moreover, $\Delta I$ for the biosensor with nanoparticles and without magnetic antibodies was 4.3 μA while $\Delta I$ for the biosensor with nanoparticles and with magnetic antibodies was 21.66 μA. Also, $\Delta I$ for the biosensor without nanoparticles and without magnetic antibodies was 4.2 μA while $\Delta I$ for the biosensor without nanoparticles and with magnetic antibodies was 10.96 μA. This indicates that the sensitivity of this biosensor can be improved by using magnetically-labeled secondary antibodies or Pt/Pd nanoparticles, or both.

**Figure 12:**
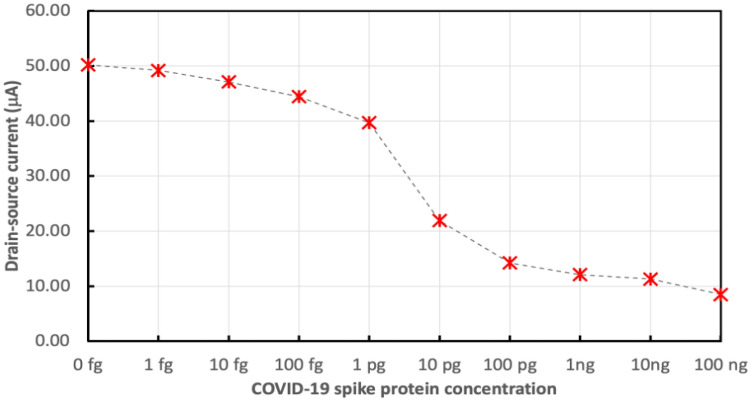
Electrical drain current for GO-FET biosensor functionalized with COVID-19 spike antibody due to 0.4 μL drop of the different concentrations of COVID-19 spike antigen.

The sensor performance is investigated toward COVID-19 spike antigen with a fixed ${V}_{ds}$(0.1 V) across the source and drain. While ${V}_{g}$ was set to zero. Figure 12 shows the drain current for GO-FET biosensor functionalized with COVID-19 spike antibody due to 0.4 μL drop of the different concentrations of COVID-19 spike antigen. The drain current (${I}_{d}$) dropped correspondingly as COVID-19 spike antigen concentration increased.

**Figure 13:**
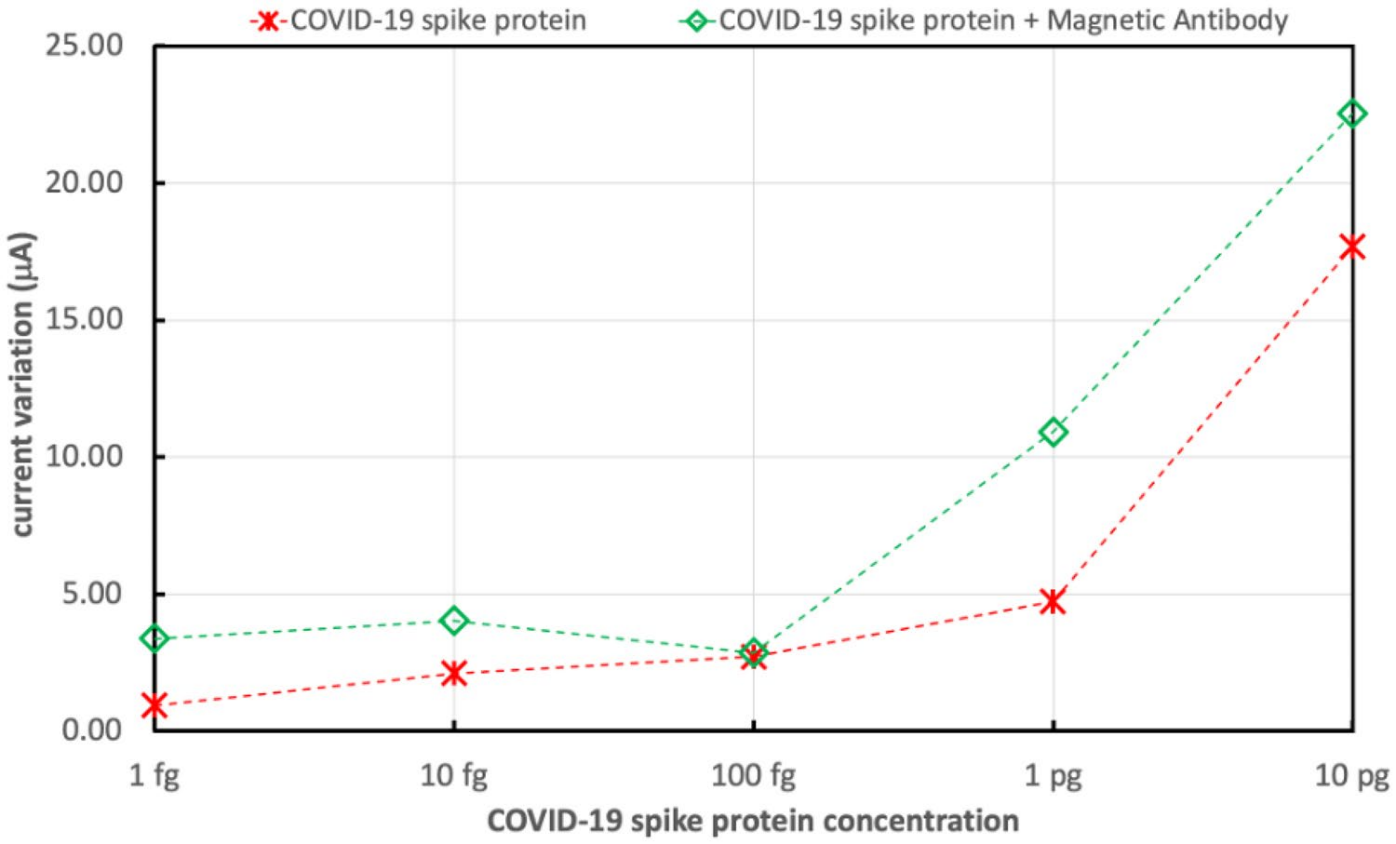
Variations in the electrical drain current of FET biosensor without nanoparticles due to different concentrations of COVID-19 spike antigen without and with the addition of magnetic antibodies.

Figure 13 shows the variations in the electrical drain current of the FET biosensor without nanoparticles due to exposure of the biosensor to different concentrations of COVID-19 spike antigen, ranging from 1 fg/mL to 10 pg/mL. Also, the sensor variation in current for the different concentrations of COVID-19 spike antigen was measured after the addition of 5 μL drop of 250 μg/mL of magnetically labeled COVID-19 spike antibodies. It was noticed that the variation in current increased and the sensor sensitivity was enhanced.

**Figure 14:**
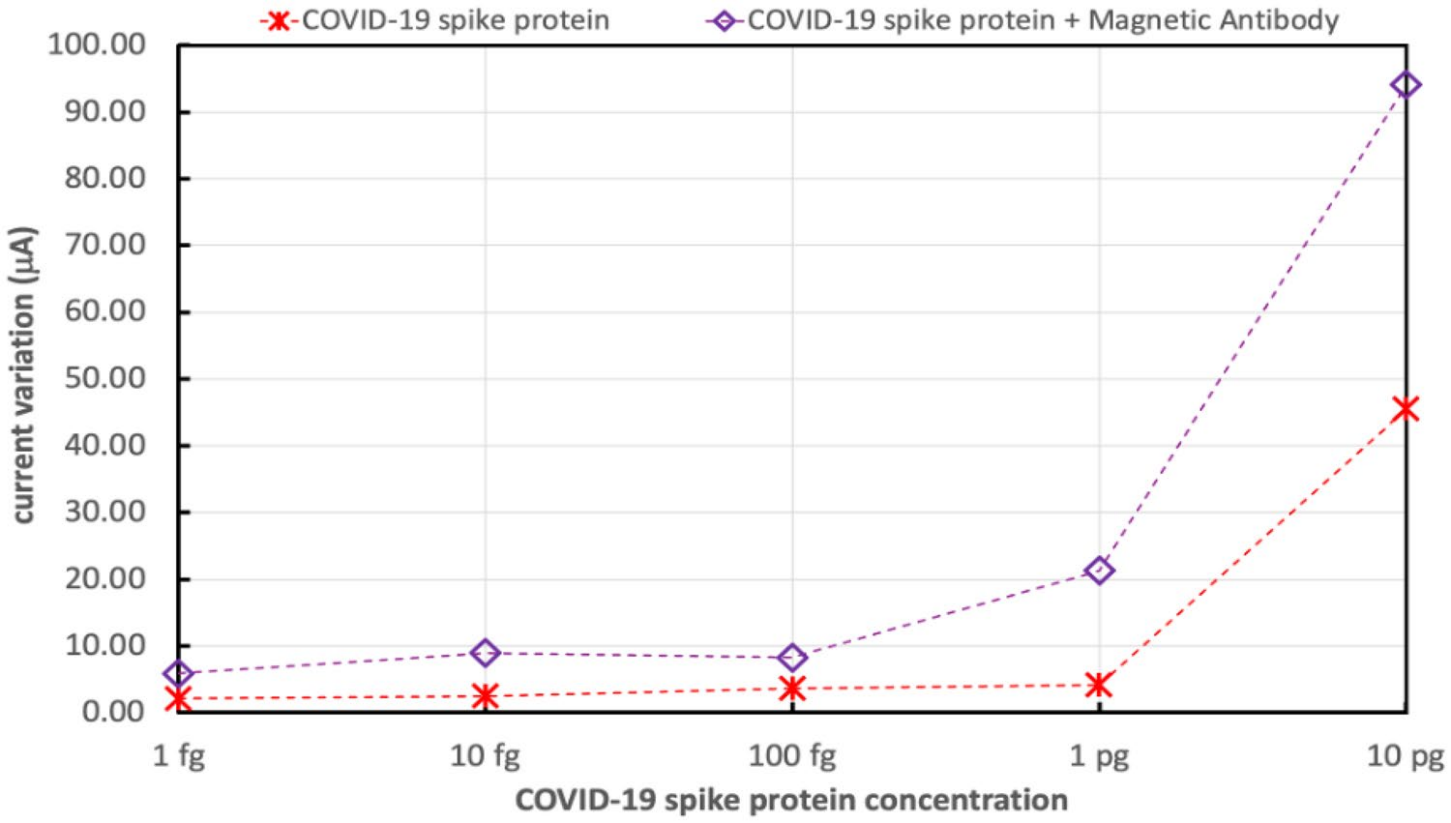
Variations in the electrical drain current of FET biosensor decorated with bimetallic Pd/Pt nanoparticles is due to the different concentrations of COVID-19 spike antigen without and with the addition of magnetic antibodies.

Figure 14 shows the variations in the electrical drain current of FET biosensor with bimetallic nanoparticles of palladium and platinum due to exposing the biosensor to different concentrations of COVID-19 spike antigen, ranging from 1 fg/mL to 10 pg/mL. Moreover, the sensor variation in current for the different concentrations of COVID-19 spike antigen was measured after the addition of 5 μL drop of 250 μg/mL of magnetically labeled COVID-19 spike antibodies.

Figures 13 and 14 show that $\Delta I$ increments are due to higher concentrations of COVID-19 spike antigen in both sensors and that the higher sensitivity for the various concentrations of COVID-19 spike antigen is due to the presence of composite bimetallic nanoparticles.

The drop in the current after the addition of the COVID-19 spike antigen is due to its negative charge which induces excess hole carriers. The more significant reduction in current for the sensor decorated with nanoparticles is due to the more adsorption of the antigen. The addition of the negative antigen leads to an increment of the holes concentration where the holes trap the electrons which increases the sensor resistance and reduces the electrical current. The current variation was more when the sensor was decorated with bimetallic nanoparticles and when the magnetic antibodies were added. The GO-FET operated as a p-type channel FET. The source and drain are made of gold (metal). The quick drop of the current confirms that the GO channel is sensitive to the transfer of electrons during antibody and antigen interaction.

Transistor based biosensor are effective platforms to acquire rapid, cheap, accurate COVID-19 virus detection^46–54^. Seo’s group developed a graphene-based field effect transistor for COVID-19 virus detection. The fabricated sensor was coated with specific protein found in COVID-19 spikes^10^. Fathi-Hafshejani et al. utilized semiconductor-based FET in in vitro sensor applications for rapid identification of COVID-19^55^. Song’s group developed an electrochemical nano-biosensor based on electropolymerized polyaniline (PANI) nanowires and newly designed peptides for COVID-19 identification ^56^. Miripour et al. developed an electrochemical sensor by taking in considering the reactive oxygen species (ROS) level as a major side effect of COVID-19 disease^57^. Various research work to detect COVID-19 are based on using electrochemical impedance spectroscopy (EIS) which is composed of various nanomaterial such as nanoparticles, graphene oxide (GO) nanocomposites, and nanodendroids^58,59^. Shan’s group was able to detect COVID-19 by analyzing patients’ breath using a sensor composed of gold nanoparticles and organic ligands^60^.Guo’s group detected COVID-19 virus by spotting the protein present in corona virus spikes by utilizing organic electrochemical transistors (OECTs)^61^. Zamzami’s group fabricated a carbon nanotube FET (CNT-FET) electrochemical nano-biosensor to detect COVID-19 spike protein (S1) in patient’s saliva^3^. In this work, real-time COVID-19 detection was achieved via GO-FET decorated with bimetallic nanoparticles of palladium and platinum with high sensitivity and selectivity.

Graphene has outstanding properties such as ultra-high mechanical strength^62^, high mobility^63,64^, and unique electrical properties^65^. Thus, graphene is considered an ideal membrane for bio-molecular transistor applications. However, it is inefficient to manufacture huge amounts of graphene membranes. Therefore, graphite oxide was utilized in this work as a promising alternative of graphene since it is more economical, has the essential sensing features, it is easier to produce, and can be easily diluted in water and used in several application such as nano-electronic devices^66^. Moreover, Noble bimetallic and trimetallic nanoclusters have attracted researchers’ interest due to the possibility to design their properties. Bimetallic and trimetallic nanoclusters have enhanced catalytic activity^67–72^, improved antimicrobial action^73^, diverse morphology^74,75^, improved antimicrobial action^73^, high sensitivity and selectivity^76–78^ and very good stability^79,80^. Therefore, bimetallic nanoparticles were utilized in our work to investigate their performance for the developed GO-FET biosensor.

## Conclusion

The GO-FET biosensors can be fabricated using a simple, low-cost methodology. Functionalization of the GO-FET biosensor with anti-COVID-19 S-protein antibody provided a rapid, simple, and responsive detection of the COVID-19 S-protein in a test solution at a concentration as low as 1 fg/mL. In contrast, no specific signals were detectable over the wide range of COVID-19 spike protein concentrations when the GO-FET biosensor was functionalized with MERS antibody. The GO-FET biosensor performance is investigated after decorating the graphite oxide channel with platinum and palladium nanoparticles. The variation in current for the GO-FET decorated with Pt/Pd nanoparticles and without the usage of magnetic antibodies was 4.3 μA while the variation in current for the GO-FET decorated with Pt/Pd nanoparticles and with the usage of magnetic antibodies was 21.66 μA. Moreover, the variation in current for the bare GO-FET and without the usage magnetic antibodies was 4.2 μA while the variation in current for the bare GO-FET and with the usage of magnetic antibodies was 10.96 μA. This indicates that the addition of the nanoparticles enhanced the sensor sensitivity to the COVID-19 spike antigen. Furthermore, the addition of magnetically labeled secondary anti-SARS-CoV-2 S-protein antibodies resulted in improved sensor sensitivity. It was also noticed that increasing the concentrations of COVID-19 spike antigen results in higher variation in current and higher sensitivity for both the bare GO-FET biosensor and for the GO-FET biosensor decorated with Pt/Pd nanoparticles. This increment in sensitivity is due to the negative charge of the COVID-19 antigen. Thus, the newly developed GO-FET biosensor provides a rapid, responsive, and simple methodology for the detection of COVID-19 spike antigen and can potentially be used for the detection of COVID-19 viruses in biological samples. This detection technology can be adapted for the detection of other types of viral and bacterial antigens, as well as biomarkers of various diseases.

## Data Availability

All data generated or analysed during this study are included in this published article.
